# Autotaxin Inhibition with PF-8380 Enhances the Radiosensitivity of Human and Murine Glioblastoma Cell Lines

**DOI:** 10.3389/fonc.2013.00236

**Published:** 2013-09-17

**Authors:** Sandeep R. Bhave, David Y. A. Dadey, Rowan M. Karvas, Daniel J. Ferraro, Rama P. Kotipatruni, Jerry J. Jaboin, Andrew N. Hallahan, Todd A. DeWees, Amanda G. Linkous, Dennis E. Hallahan, Dinesh Thotala

**Affiliations:** ^1^Department of Radiation Oncology, Washington University in Saint Louis, St. Louis, MO, USA; ^2^School of Medicine, Washington University in Saint Louis, St. Louis, MO, USA; ^3^NYU Cancer Institute, New York University Langone Medical Center, New York, NY, USA; ^4^Mallinckrodt Institute of Radiology, Washington University in Saint Louis, St. Louis, MO, USA; ^5^Siteman Cancer Center, Washington University in Saint Louis, St. Louis, MO, USA; ^6^The Hope Center, Washington University in Saint Louis, St. Louis, MO, USA

**Keywords:** glioblastoma, radiosensitizer, autotaxin, lysophosphatidic acid, PF-8380

## Abstract

**Purpose:** Glioblastoma multiforme (GBM) is an aggressive primary brain tumor that is radio-resistant and recurs despite aggressive surgery, chemo, and radiotherapy. Autotaxin (ATX) is over expressed in various cancers including GBM and is implicated in tumor progression, invasion, and angiogenesis. Using the ATX specific inhibitor, PF-8380, we studied ATX as a potential target to enhance radiosensitivity in GBM.

**Methods and Materials:** Mouse GL261 and Human U87-MG cells were used as GBM cell models. Clonogenic survival assays and tumor transwell invasion assays were performed using PF-8380 to evaluate role of ATX in survival and invasion. Radiation dependent activation of Akt was analyzed by immunoblotting. Tumor induced angiogenesis was studied using the dorsal skin fold model in GL261. Heterotopic mouse GL261 tumors were used to evaluate the efficacy of PF-8380 as a radiosensitizer.

**Results:** Pre-treatment of GL261 and U87-MG cells with 1 μM PF-8380 followed by 4 Gy irradiation resulted in decreased clonogenic survival, decreased migration (33% in GL261; *P* = 0.002 and 17.9% in U87-MG; *P* = 0.012), decreased invasion (35.6% in GL261; *P* = 0.0037 and 31.8% in U87-MG; *P* = 0.002), and attenuated radiation-induced Akt phosphorylation. In the tumor window model, inhibition of ATX abrogated radiation induced tumor neovascularization (65%; *P* = 0.011). In a heterotopic mouse GL261 tumors untreated mice took 11.2 days to reach a tumor volume of 7000 mm^3^, however combination of PF-8380 (10 mg/kg) with irradiation (five fractions of 2 Gy) took more than 32 days to reach a tumor volume of 7000 mm^3^.

**Conclusion:** Inhibition of ATX by PF-8380 led to decreased invasion and enhanced radiosensitization of GBM cells. Radiation-induced activation of Akt was abrogated by inhibition of ATX. Furthermore, inhibition of ATX led to diminished tumor vascularity and delayed tumor growth. These results suggest that inhibition of ATX may ameliorate GBM response to radiotherapy.

## Introduction

Glioblastoma multiforme (GBM), the most common primary brain tumor, is a highly aggressive and universally fatal cancer (1). The diffuse, invasive, and highly angiogenic characteristics that define glioblastoma result in a high recurrence rate, despite high-dose radiation therapy with wide margins (1–3). Following surgical resection, the current standard of care involves administering the DNA alkylating agent temozolomide (TMZ) concurrently with irradiation, followed by adjuvant TMZ (4). While this treatment regimen does confer a survival advantage, overall survival of patients with GBM remains abysmal at around 1 year. Furthermore, patients without MGMT methylation only have a marginal benefit with TMZ (4). The development of molecular targeted therapy that increases the efficacy of radiation therapy would potentially allow for improved local control and improved outcomes in the treatment of GBM.

Targeting mitogenic pathways to radiosensitize GBM is under vigorous investigation. Preclinical studies have shown vascular endothelial growth factor receptor (VEGFR) signaling to contribute to the highly angiogenic nature of GBM (5) and VEGF up-regulation occurs following irradiation (6, 7). Accordingly, several drugs have targeted VEGF signaling using traps, aptamers, and antibodies in an effort to radiosensitize GBM. Aflibercept, a VEGF Trap, was shown to delay tumor growth in preclinical mouse models of GBM when combined with irradiation (8). The VEGF aptamer pegaptanib improved survival when combined with whole brain irradiation in orthotopic mouse models for GBM (9). VEGF inhibition with antibodies has also shown to delay tumor growth in a greater than additive fashion in several human cell lines in orthotopic mouse tumor models, including human glioblastoma cell lines (7). In clinical trials, Bevacizumab, a monoclonal antibody to VEGF, improves survival temporarily, but the benefit is short lived (10). Furthermore, preliminary results of the Phase III trial RTOG 0825 showed no improvement in overall survival when bevacizumab was added to standard chemoradiotherapy following resection in patients with newly diagnosed GBM. Despite these disappointing results, bevacizumab is one of the few drugs that has shown efficacy in recurrent GBM (11). Drugs targeting DNA repair are also under investigation. Irinotecan, a topoisomerase I inhibitor, while having promising radiosensitization in preclinical studies, has shown marginal improvements in survival in clinical trials (12). ATM Kinase inhibition has also been shown to radiosensitize glioblastoma in early preclinical studies (13).

Small molecule protein kinase inhibitors are also being actively investigated as GBM radiosensitizers. Vatalanib (PTK787) inhibits VEGF, platelet derived growth factor (PDGF), and c-kit, and has been shown to delay tumor growth when combined with irradiation in a colorectal cancer animal model (14). This drug is being moved to clinical studies, showing an acceptable toxicity profile in recently completed phase I trials in GBM (15). Enzastaurin, a protein kinase C inhibitor with anti-angiogenic activity, has been shown to delay tumor progression in animal models of GBM and is showing encouraging results in ongoing clinical trials (16, 17).

A recent genetic analysis from the Cancer Genome Atlas revealed that 88% of GBM has somatic alterations in the receptor tyrosine kinase (RTK)/RAS/PI3K pathway (18). Gefitinib, a drug targeting epidermal growth factor receptor (EGFR), the most commonly mutated RTK, is being investigated as a radiosensitizer in clinical trials. Unfortunately, early clinical results have been disappointing. Recent early phase trials from the RTOG and Mayo/North American Cancer Treatment group have shown no survival benefit in patients receiving gefitinib with irradiation when compared to historic controls (19, 20).

The Akt/Protein Kinase B (PKB) pathway is a central downstream target of the RTK/RAS/PI3K pathway. The Akt pathway has been implicated in several cancers and supports a myriad of cellular activities including angiogenesis, cell survival, proliferation, and migration (21). Elevated Akt and phosphorylated Akt have not only been demonstrated in several glioblastoma cell lines and tissue samples, but have also shown to be associated with radioresistance (22, 23). Indeed, pharmacological inhibition of Akt has been shown to increase radiosensitivity of glioblastoma *in vitro* (23, 24). We have previously shown that irradiation of tumor endothelium leads to increased production of cytosolic phospholipase A2 (cPLA2), which in turn catalyzes the production of lysophosphatidylcholine (LPC) (24). LPC can function as a secondary messenger in a variety of signaling pathways, including the Akt/PKB pathway (25, 26). The activation of Akt leads to radioresistance within tumor vascular endothelium and hinders the efficacy of radiotherapy (25–28).

Autotaxin (ATX), an enzyme with lysophospholipase D (lysoPLD) activity, catalyzes the production of lysophosphatidic acid (LPA) from LPC (29–31). ATX is a 125 kDa autocrine tumor motility enzyme and is a member of the ectonuclease pyrophosphatase/phosphodiesterase (NPP) family. ATX not only possesses a lysoPLD activity, it also is a lipid carrier protein that efficiently transports LPA to respective cognate LPA_1–6_ GPCRs (32). Accumulating evidence points to ATX and LPA playing a role in tumor progression, invasion, and angiogenesis (33, 34). ATX is highly expressed in a variety of cancers including non-small cell lung cancer (NSCLC) (35), ovarian cancer (36), breast cancer (37), and GBM (38, 39). In GBM, ATX is preferentially expressed in actively invading tumor cells (39). ATX overexpression in GBM is believed to facilitate invasion and migration through endothelial cells in an autocrine fashion as well as promote neovascularization in the tumor core through paracrine signaling (2, 34).

Alpha-bromomethylene phosphonate (Brp-LPA), a pan LPA receptor and ATX inhibitor, was shown to significantly enhance radiation-induced cell death and disrupt cell invasion, cell migration, and pro-survival pathways (27, 28). Pre-treatment with Brp-LPA prior to irradiation was shown to significantly enhance radiation-induced cell death and disrupt cell invasion, cell migration, and pro-survival pathways.

Our previous work led us to hypothesize that inhibition of ATX alone could effectively radiosensitize glioblastoma through decreased production of LPA. We studied the effect of non-lipid small molecule ATX inhibitor PF-8380 (40) in glioblastoma cells and tumor vascular endothelial cells, using murine and human cell lines. We found that inhibition of ATX enhances radiation induced cell death and disrupts Akt signaling in both glioblastoma and tumor vascular endothelial cells. We also found that inhibition of ATX inhibits migration, and decreases invasion in glioma cell lines. Most importantly, we found that pre-treatment with PF-8380 prior to irradiation inhibited radiation-induced angiogenesis of tumor vascular endothelial cells and delayed progression of glioma tumor growth *in vivo*. Our findings suggest that inhibition of ATX alone could serve as a potential treatment strategy to increase the effectiveness of radiotherapy in glioblastoma.

## Materials and Methods

### Cell cultures and treatments

Human glioblastoma (U87-MG) cells and mouse brain microvascular (bEnd.3) cells were obtained from ATCC and maintained in RPMI 1640 with 10% fetal bovine serum (FBS) and DMEM with 10% FBS respectively. Human Umbilical Vein Endothelial (HUVEC) cells were obtained from Lonza and were maintained in EGM2 media (Lonza, USA). Mouse glioma GL261 cell lines were obtained from Dr. Yancie Gillespie (University of Alabama, Birmingham, AL, USA) and maintained in DMEM with Nutrient Mixture F-12 1:1, 10% FBS, 1% sodium pyruvate (Life Technologies, USA). All cells were grown in a 5% CO2 incubator at 37°C. ATX inhibitor PF-8380 (40) [6-(3-(piperazin-1-yl) propanoyl)benzol(d)oxazol-2(3*H*)-one] was obtained from Pfizer Inc. under the Pfizer–Washington University biomedical agreement. The concentration of PF-8380 was calculated from the data concerning the kinetics of inhibition of LysoPLD activity of purified ATX (40). For the radiation of cells and mice, RS2000 (Rad Source Technologies, Inc., USA) a 160 kV x-ray machine with a 0.3 mm Cu filter was used.

### Quantitative real-time PCR analysis

RNA was harvested using TRIZOL reagent per manufacturer’s protocol (Invitrogen, Carlsbad, CA, USA). Total RNA was then purified using the RNeasy Mini Kit (Qiagen, USA). RNA was reverse transcribed using the high capacity cDNA Reverse Transcriptase kit (Invitrogen, USA) according to the manufacturer’s protocol. Single stranded cDNA products were then analyzed by real-time PCR using standard commercially available TaqMan probes from Applied Biosystems for mouse (Mm00516572_m1) or human ATX (Hs00905125_m1) gene. Housekeeping genes of mouse (Mm99999915_g1) or human GAPDH (Hs02758991_g1) were used in the TaqMan Gene Expression Assay to normalize any possible variations for the target ATX gene. Delta–delta *C*_t_ relative gene expression analysis was employed and results were compared to either expression from U87-MG in human lines or GL261 in mouse samples. Mean expression and standard error were calculated for each group. PCR products were also run on a 1.8% agarose gel in 1× TAE buffer stained with ethidium bromide and visualized under UV light.

### Co-culture clonogenic survival assay

HUVEC (1.0 × 10^6^) and bEnd.3 cells (1.0 × 10^6^) were plated in 100 mm plates and after 24 h, U87-MG (2 × 10^6^) and GL261 (2 × 10^6^) cells were plated onto transwell inserts (Corning Inc., USA). After co-culture for 24 h, cells were treated with 1 μM of PF-8380 or vehicle control DMSO for 45 min prior to irradiation with 0, 2, 4, 6, or 8 Gy. After the treatments as co-culture with either PF-8380 or DMSO calculated numbers of U87-MG and GL261 cells were plated to enable normalization for plating efficiencies. After 7 to 10 day incubation plates were fixed with 70% EtOH and stained with 1% methylene blue. Colonies consisting of>50 cells were counted by viewing the plates under a microscope. The survival fractions were calculated as (number of colonies/number of cells plated)/(number of colonies for corresponding control/number of cells plated). Survival curves were analyzed by curve fitting to the alpha/beta model (41) calculating *D*_0_ and *n*.

### Wound healing/scratch assay for cell migration

GL261 or U87-MG cells were plated in triplicate onto 6 cm plates and allowed to grow to 70% confluence. The semi-confluent cell layer was scratched with a sterile 200 μL pipette tip to create a scratch devoid of cells and plates were washed once with PBS to remove non-adherent cells and debris. For radiosensitization drug studies, cells were treated with 1 μM PF-8380 or DMSO for 45 min prior to irradiation with 4 Gy, and then incubated at 37°C in 5% CO_2_. Control plates were monitored for cell migration (20–24 h). Cells were fixed with 70% ethanol and stained with 1% methylene blue. To quantify migration, cells in three randomly selected high power fields (HPFs) in the scratched area were counted and normalized for surrounding cell density. Mean and standard error for each treatment group were calculated.

### Tumor transwell-invasion assays

The tumor transwell matrigel invasion assay has previously been used to aid in quantitation of the tumor endothelium interactions and transmigrations (42). GL261 (1.0 × 10^6^ cells/well) or U87-MG (0.6 × 10^6^ cells/well) were suspended in serum-free media and added onto the upper chamber (inserts) that was matrix-coated polycarbonate membrane filters with 8 μm pores (Cell Biolabs Inc., USA). Five hundred microliters of fresh medium was added to the bottom chamber. For radiosensitization drug studies, both chambers were then treated with vehicle DMSO or 1 μM PF-8380 for 45 min prior to irradiation with 4 Gy. After 36 h, remaining cells in the upper chamber of the membrane inserts were removed using a wet cotton swab. The cells that adhered on the outer surface of the transwell insert membrane which had invaded through the matrigel were fixed with 100% methanol, and stained. Invaded cells in 7–10 HPF from each sample were counted using Image J Software (NIH, Bethesda, MD, USA), and the average number of invaded cells per HPF was calculated. Mean and standard error for each treatment group were calculated.

### Immunoblot analysis

To analyze the expression of extracellular ATX protein from endothelial and glioma cells, conditioned media from HUVEC, bEnd.3, GL261, and U87-MG cells were collected and subsequently concentrated using centrifugal filter concentrators (Amicon). The concentrated media containing the extracellular ATX was immunoblotted using a specific antibody to ATX (Santa Cruz, Biotechnology, USA). In mouse and human co-culture studies, bEnd.3 cells or HUVEC (1 × 10^6^) were plated in 10 cm well plates and after 24 h, GL261 or U87-MG (2 × 10^6^) cells were plated onto transwell inserts (Corning Inc., USA). After the treatments total protein was extracted from treated cells using the M-PER mammalian protein extraction reagent (Pierce, Rockford, IL, USA). Protein concentration was quantified using BCA Reagent (Pierce, Rockford, IL, USA) and protein extracts were analyzed using specific antibodies to phospho-Akt^T308/S473^ and total Akt (Cell Signaling Technologies, Danvers, MA, USA). Antibody to actin (Sigma, USA) was used to evaluate protein loading in each lane. Immunoblots were developed using the Western Lightning Chemiluminescence Plus detection system (PerkinElmer, USA) according to the manufacturer’s protocol. Band densities were quantitated using Image J software (NIH, Bethesda, MD, USA).

### *In vivo* angiogenesis assay dorsal skin-fold chamber model

The implantation technique of the dorsal skin-fold chamber model has been described previously (43). Briefly, diffusion chambers containing GL261 cells (1 × 10^6^ cells per chamber) were inserted in the dorsal air sac made by making a superficial incision horizontally along the edge of the dorsal air sac. The skin was carefully sutured after placing the chambers underneath the skin of each mouse. The mice treatments were performed 5–7 days following surgical insertion of the diffusion chambers. The skin fold covering the chambers was carefully removed after euthanizing the mice and photographed under visible light. The number of tumor induced blood vessels was counted in six to eight different fields within the chamber in the area of the air sac fascia.

### Mice, treatment, and tumor growth delay

All animal procedures used in this study were approved by IACUC. Handling of animals and housing was followed as per DCM guidelines. GL261 cells (1 × 10^6^) were injected into the right hind limb of nude mice. Once tumors were palpable the mice were serpentine sorted into groups of six to seven animals representing similar distributions of tumor sizes (range = 240 mm^3^). Tumor bearing mice were injected intraperitoneally with vehicle (DMSO) or PF-8380 at 10 mg/kg body weight once daily for five consecutive days. Forty five minutes after drug injection, mice were anesthetized with isoflurane and positioned in the RS2000 irradiator. They were then irradiated with 2 Gy daily for five consecutive days for a total of 10 Gy. Lead blocks (10 mm thick) were used to shield the head, thorax, and abdomen. Tumor size was monitored longitudinally using an external traceable digital caliper (Fisher Scientific, USA). Mice were sacrificed by cervical dislocation once the tumors reached a volume of approximately 10 mm^3^ or when ulceration became apparent on the hind limb per Animal Care guidelines.

### Statistical analyses

The mean and standard error of the mean (SEM) of each treatment group were calculated for all experiments. The number of samples is indicated in the description of each experiment. Statistical analysis was done using a student’s *t*-test to compare two means with *P* < 0.05 representing statistical significance. Non-parametric Kruskal–Wallis test was used to compare treatment effectiveness in the tumor growth delay experiment. All pair-wise comparisons between treatment groups were adjusted using Tukey’s multiple comparison method. A *P*-value of<0.05 was considered statistically significant.

## Results

### ATX is highly expressed in U87-MG and GL261 glioblastoma cell lines

**Figure 1:**
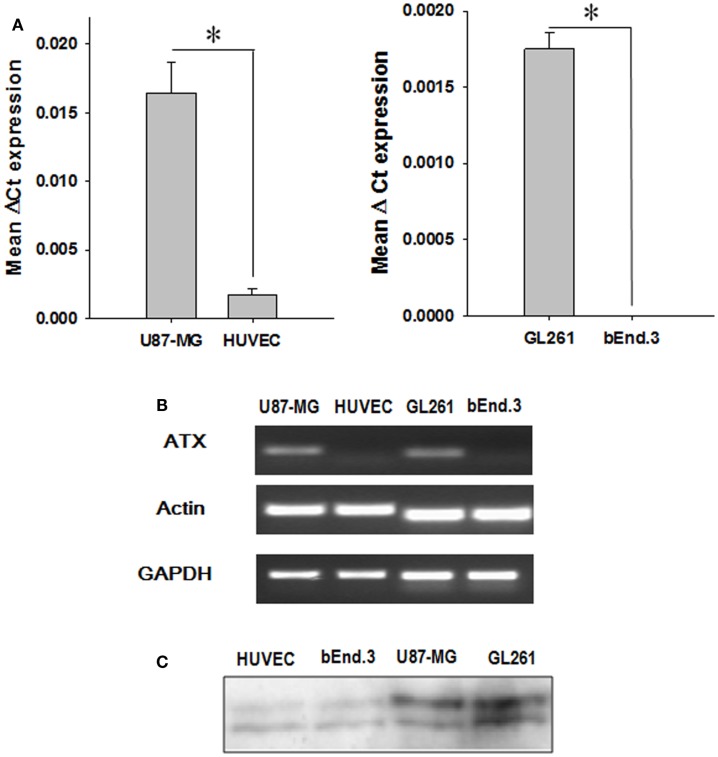
**ATX expression in endothelial and GBM cell lines**. **(A)** The mRNA from endothelial cells (HUVEC and bEnd.3) as well as tumor cells (U87-MG and GL261) were analyzed by quantitative PCR using TaqMan probes specific for ATX. The bar graph showing the mean Δ*C*_t_ values of expression of ATX normalized to actin is shown. **(B)** A representative gel indicating the mRNA levels of ATX, actin, and GAPDH is shown. **(C)** The conditioned medium from endothelial cells (HUVEC and bEnd.3) and tumor cells (U87-MG and GL261) was concentrated and then were immunoblotted and probed with an anti ATX antibody.

We analyzed the expression of ATX mRNA in GBM cell lines (U87-MG and GL261) and endothelial cell lines (HUVEC and bEnd.3) by quantitative real-time polymerase chain reaction (Q-PCR). Examination of the mean Δ*C*_t_ values revealed significantly higher levels of ATX expression in glioblastoma cells relative to the corresponding endothelial cell models (U87-MG, *P* < 0.001; GL261, *P* = 0.002) (Figure 1A). This result was confirmed further by analyzing the PCR products on a 2% agarose gel (Figure 1B). Quantitative analysis of the gel showed significantly higher levels of ATX expression in glioblastoma cells relative to the corresponding endothelial cell models (U87-MG, *P* < 0.001; GL261, *P* = 0.002). ATX was first characterized as a 125 kDa protein in the conditioned medium of human melanoma cells (44). We probed the concentrated conditioned media of U87-MG, GL261, HUVEC, and bEnd.3 cells by western immunoblots for extracellular ATX. Extracellular ATX was found at higher levels in the glioblastoma conditioned media when compared to endothelial cell conditioned media (Figure 1C).

### Inhibition of ATX enhances radiation-induced cell death in irradiated U87-MG and GL261

**Figure 2:**
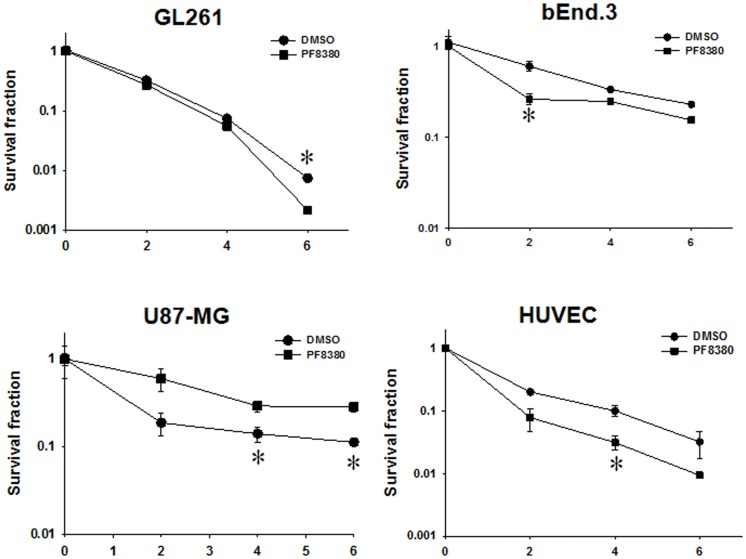
**Inhibition of ATX enhances radiation-induced cell death in irradiated GBM cells (U87-MG and GL261) in co-culture with endothelial cells (HUVEC and bEnd.3) monitored by clonogenic survival assays**. Human U87-MG and murine GL261 glioma cells were plated in co-culture with corresponding HUVEC and bEnd.3 endothelial cells. Cells were treated with 1 μM PF-8380 (■) or DMSO (•) for 45 min and irradiated with 0, 2, 4, and 6 Gy. The cells were incubated for 7–10 days and colonies were fixed, stained with 1% methylene blue, and colonies greater than 50 cells were counted. Shown are the clonogenic survival curve and mean surviving fractions and SEM; *P > 0.05.

To determine if inhibition of ATX is sufficient to enhance radiosensitivity of glioblastoma cells, we tested the efficacy of ATX inhibitor PF-8380 in co-culture clonogenic assays designed to simulate the tumor microenvironment. U87-MG and GL261 cells were grown in co-culture with HUVEC and bEnd.3 respectively and treated with 1 μM PF-8380 in media containing lipid-free BSA 45 min prior to irradiation (Figure 2). The concentration of PF-8380 was calculated from the data concerning the kinetics of inhibition of LysoPLD activity of purified ATX (40). Pre-treatment of GL261 and bEnd.3 with PF-8380 resulted in decreased cell survival compared to cells treated with radiation alone (GL261: 6 Gy, *P* = 0.005; bEnd.3: 2 Gy, *P* = 0.012, 4 Gy, *P* = 0.001, 6 Gy, *P* = 0.003). Similar results were found for U87-MG and HUVEC, where pre-treatment with PF-8380 also lead to a significant decrease in cell survival relative to cells treated with radiation alone (U87-MG: 4 Gy, *P* = 0.038, 6 Gy, *P* = 0.008; HUVEC: 4 Gy, *P* = 0.033). The curves were fitted and analyzed using the alpha/beta model (41). There were no significant changes in the value of the extrapolation numbers (*n*). The *D*_0_ values decreased in all four cell types that were irradiated, confirming the conclusion reached from the analysis of survivals at different doses, as described above. In the mouse glioblastoma cells, the *D*_0_ decreased from 1.03 to 0.84 Gy, while in the human cells, it decreased from 2.3 to 1.85 Gy. In the endothelial cells, in the mouse bEnd.3 cells the *D*_0_ decreased from 3.62 to 2.86 Gy, while in the HUVEC cells, it decreased from 1.70 to 1.19 Gy.

### Inhibition of ATX attenuates cell migration and cell invasion in irradiated GBM cells

**Figure 3:**
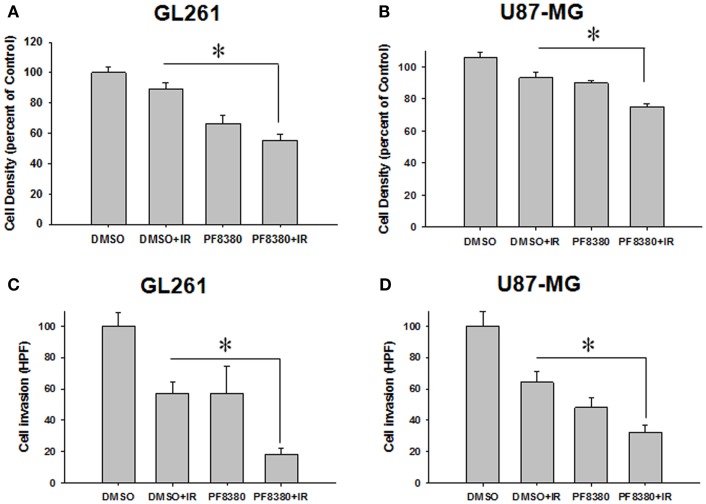
**Inhibition of ATX attenuates cell migration in irradiated GBM cells**. Human glioma U87-MG **(A)** and mouse glioma GL261 cells **(B)** were plated on 60 mm plates and allowed to grow to 70% confluency. A gash was created, washed in PBS, and treated with vehicle (DMSO) or 1 μM PF-8380 for 45 min before irradiation with 4 Gy. After 24 h, cells were fixed in 70% EtOH and stained with methylene blue. Migrated cells were counted and normalized to surrounding cell density per HPF. Shown are the bar graphs **(A,B)** representing the mean percentages of migrating cells relative to corresponding controls with SEM; *P > 0.05. Inhibition of autotaxin attenuates cell invasion in irradiated glioblastoma cells. GL261 **(C)** and U87-MG **(D)** cells were added to the eight micron inserts and were treated with 1 μM PF-8380 or DMSO for 45 min prior to irradiation with 4 Gy. Cells were allowed to invade into the complete medium at the bottom of the inserts for 48 h. Cells were fixed, stained, and cell invasion was calculated by counting the number of cells per HPF. Shown are the bar graphs representing the number of invasive cells with SEM; *P > 0.05.

Since ATX overexpression has been previously implicated in enhancing the invasive and migratory characteristics of glioblastoma cells (38, 40), we examined the effect of ATX inhibition by PF-8380 in GL261 and U87-MG cell lines. In scratch assays performed for GL261 we observed an 11.1% decrease in cell migration when cells were irradiated with 4 Gy alone; however pre-treatment with 1 μM PF-8380 resulted in a 33% further reduction in cell migration (*P* = 0.002) (Figure 3A). Similarly in U87-MG, radiation alone caused a 6.1% reduction in migration, while pre-treatment with 1 μM PF-8380 reduced migration further by 17.9% (*P* = 0.012) (Figure 3B). In transwell-invasion assays, we found a significant decrease in invasion by 50% in GL261 after cells were irradiated with 4 Gy. However, the addition of PF-8380 prior to irradiation resulted in an additional decrease in invasion by 35.6% (*P* = 0.0037) (Figure 3C). Similar results were observed in U87-MG, where radiation alone caused a 35.6% reduction in invasion, while pre-treatment with PF-8380 further reduced invasion by 31.8% (*P* = 0.002) (Figure 3D).

### Inhibition of ATX disrupts Akt signaling in GBM cells

**Figure 4:**
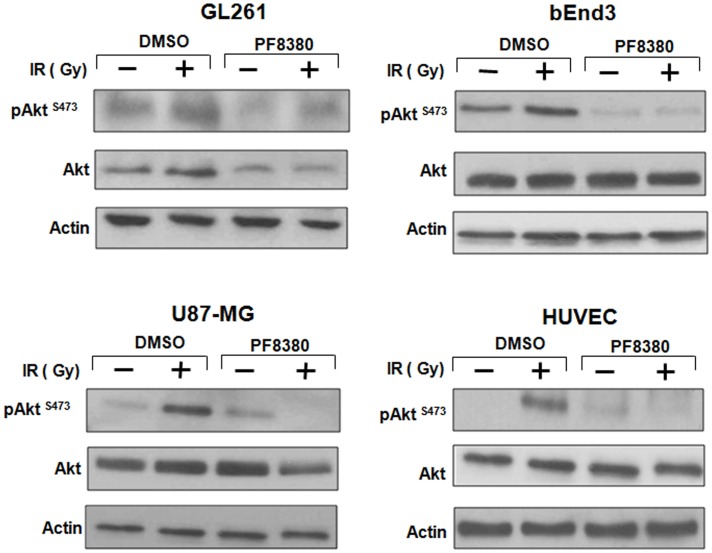
**Inhibition of ATX reduces Akt Phosphorylation in GBM cells grown in co-culture**. U87-MG and GL261 glioma cells were plated in co-culture with corresponding HUVEC and bEnd.3 endothelial cells. Cells were treated with 1 μM PF-8380 or DMSO for 45 min and irradiated with 4 Gy. Cells were lysed 15 min after irradiation and cellular proteins were immunoblotted using antibodies against pAkt^Ser473/Thr308^, total Akt, to analyze levels of expression of these proteins. Actin was used to evaluate the protein loading in each lane.

ATX may activate pro-survival pathways like Akt following irradiation through the production of LPA. Earlier, we have shown that inhibition of both ATX and LPA receptors with Brp-LPA diminished Akt phosphorylation in irradiated glioblastoma and endothelial cell lines grown in co-culture (28). We investigated whether inhibition of ATX alone with PF-8380 could abrogate Akt phosphorylation following irradiation. Glioblastoma (GL261 or U87-MG) cells were grown in co-culture with endothelial cells (bEnd.3 or HUVEC). These co-cultures were then treated with 1 μM PF-8380 or vehicle control for 45 min prior irradiation with 4 Gy. Glioblastoma and endothelial cells treated with 4 Gy showed increased Akt activity (pAkt Ser-473) relative to Akt in both lines. Treatment with 1 μM PF-8380 prior to 4 Gy, however, reduced Akt phosphorylation in both glioblastoma and endothelial cell lines relative to treatment with 4 Gy alone (Figure 4). With the exception of GL261 where total Akt levels decreased with pre-treatment of PF-8380 without and with irradiation, Akt and actin levels were relatively unaffected across all four cell lines.

### Inhibition of ATX abrogates radiation-induced tumor neovascularization in the window model

**Figure 5:**
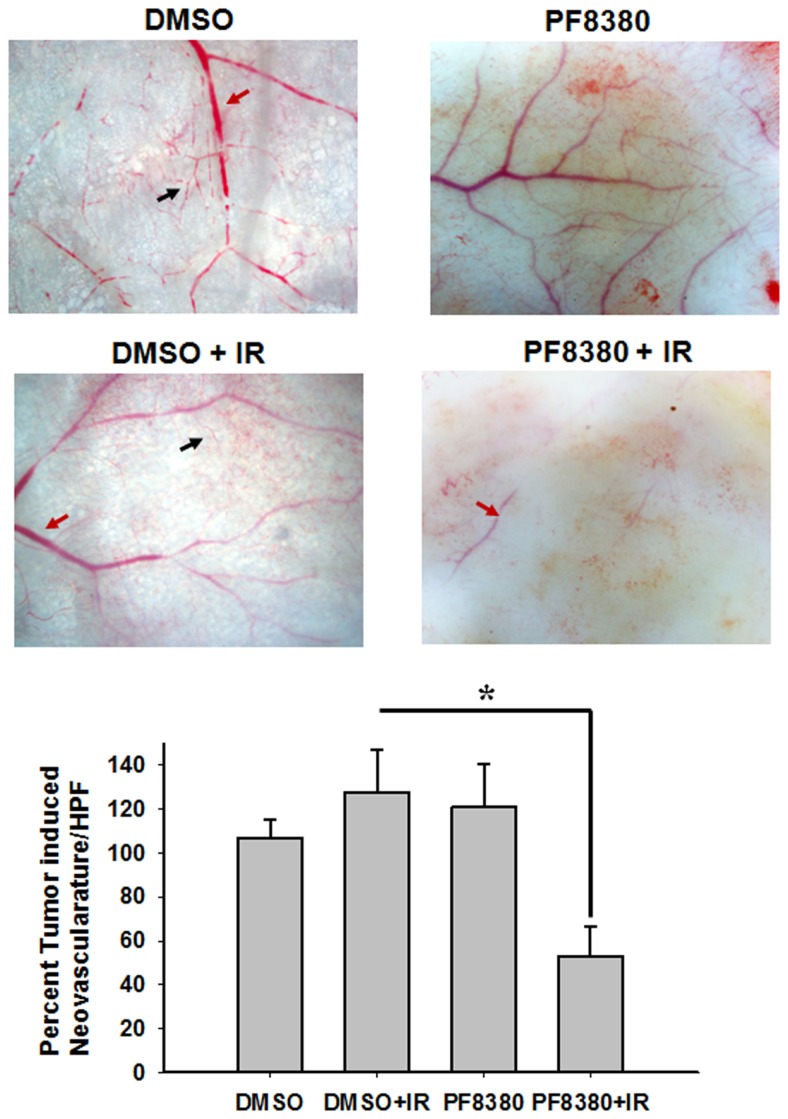
**Inhibition of ATX abrogates radiation induced tumor neovascularization**. The animals were implanted with diffusion chambers containing GL261 cells. Seven days after implantation, the mice were treated with 10 mg/Kg PF-8380 or DMSO for 30 min prior to irradiation (3 Gy). After 14 days of implantation, the animals were sacrificed and the skin fold covering the diffusion chamber was observed. Shown are the representative micrographs showing the tumor induced neovasculature (black arrow) and preexisting vasculature (red arrow) and the bar graph depicting the mean number of neovasculature (>10 field for each mouse) SEM from each treatment group of five mice; *P < 0.05.

ATX has been implicated in angiogenesis, with the enzyme linked to the up-regulation of pro-angiogenic factors, such as VEGF (45). To study the effect of inhibition of ATX on tumor-associated angiogenesis, a dorsal window model experiment was performed. Following 4 Gy irradiation, tumor-associated vascularity increased 27% (*P* = 0.382; Figure 5). Treatment with 10 mg/kg PF-8380 increased tumor-associated vascularity modestly by 20% (*P* = 0.497). When compared to control, treatment of PF-8380 45 min before 4 Gy irradiation decreased vascularity by nearly 48% when compared to control (*P* = 0.031) and by 65% when compared to mice that received radiation alone (*P* = 0.011).

### Inhibition of ATX delays tumor growth in irradiated GL261 mouse model

**Figure 6:**
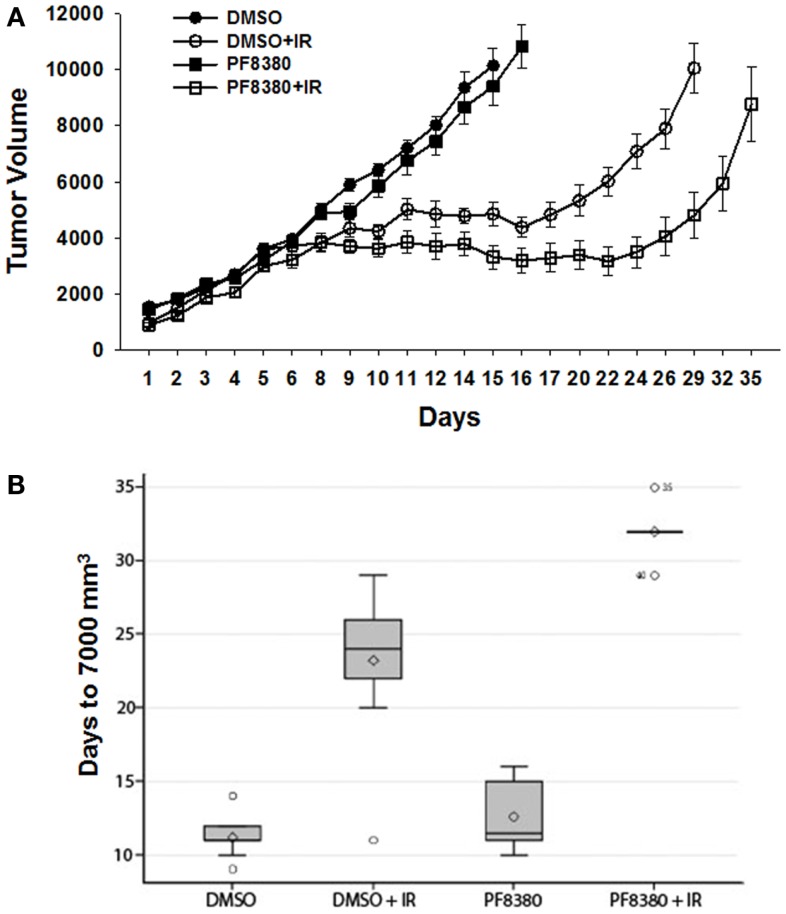
**Inhibition of ATX in combination with irradiation delays tumor growth in a heterotopic tumor model of GL261**. GL261 cells were implanted into the hind limbs of nude mice. Mice were treated for five consecutive days with PF-8380 or vehicle control prior to irradiation. Tumors were irradiated with 2 Gy for five consecutive days for a total of 10 Gy. Shown is a line graph depicting the mean tumor volumes with SEM from each treatment group of 10 mice *p < 0.05 **(A)**. The box plot depicting the tumor growth delay calculated as the number of days for tumors to reach 7000 mm^3^ **(B)**.

To evaluate the efficacy of ATX inhibitor PF-8380 *in vivo*, a heterotopic GL261 mouse tumor model was used. GL261 cells (1 × 10^6^) were injected subcutaneously in the right flank of the Nu/Nu mice. Once the tumors were palpable, the tumor-bearing mice were serpentine sorted so that the average tumor volumes were the same in all the four treatment groups. Seven tumor bearing mice were used for each treatment. Mice were either treated with vehicle (DMSO) alone, irradiation alone (five fractions of 2 Gy daily), 10 mg/kg PF-8380 alone, or a combination of 10 mg/kg PF-8380 with irradiation (five fractions of 2 Gy). The endpoint for analysis was based on the number of days taken to reach a tumor volume of 7000 mm^3^. Untreated mice reached this endpoint after 11.2 days, while PF-8380 treated mice and mice treated with radiation alone exhibited delayed tumor growth of 12.6 and 23.2 days, respectively. Furthermore, mice treated with a combination of PF-8380 and radiation required more than 32 days before reaching the endpoint tumor volume (Figures 6A,B). Kruskal–Wallis and Tukey’s pairwise comparisons showed a significant difference in number of days taken to reach a tumor volume 7000 mm^3^ between irradiation treated mice and mice treated with a combination treatment of PF-8380 and irradiation (*P* < 0.05).

## Discussion

Multi-modal therapeutic strategies that integrate aggressive de-bulking surgery with high dose radiation in combination with TMZ have improved outcomes in glioblastoma. However the radioresistance of these tumors renders these approaches ineffective in halting progression and recurrence in the majority of patients (44). Glioblastoma remains highly resistant to radiation therapy due to its mitogenic, migratory, invasive, and angiogenic characteristics. The enhanced expression of ATX in glioblastomas has been cited as a major contributor to its invasive properties, which are mediated by the paracrine actions of the LPA produced by ATX (38). The discovery that radiation can trigger the activation of ATX and LPA signaling provides a potential explanation for the behaviors of glioblastoma in the therapeutic response to radiation therapy (27). Extracellular secretion of ATX from cancer cells following irradiation leads to conversion of LPC to LPA due to its lysoPLD activity. LPA then binds to G-protein coupled receptors (GPCRs), known as (46) LPA_1–6_ which function to promote events such as cell survival and proliferation, especially in the nervous, vascular, immune, and reproductive system, while also promoting tumor invasion and tumor angiogenesis (38).

We have recently shown that inhibition of both ATX and LPA receptors by Brp-LPA leads to inhibition of migration and tubule formation in endothelial cells (28). Brp-LPA also repressed the growth of GL261 in a heterotopic mouse tumor model. To further characterize the relationship between ATX and tumor behavior independent of LPA receptor blockade, we sought to determine if inhibition of ATX alone would be sufficient to stall tumor growth and progression. We used PF-8380, a specific chemical inhibitor of ATX that has been shown to successfully inhibit lysoPLD enzyme activity in various inflammatory conditions, including cancer, multiple sclerosis, and arthritis (40). In this study, we found that inhibition of ATX by PF-8380 can significantly alter the glioblastoma microenvironment, thereby abrogating the resistant and invasive properties of glioblastoma and improving its response to radiation therapy.

The rationale for using ATX as a potential therapeutic target is based on reports that ATX is over expressed in various cancers including glioblastoma (38), lung cancer (47, 48), and breast cancer (49), and that its activity contributes to its invasiveness and tumorigenesis. To establish the significance of ATX in gliomblastoma models, we evaluated the expression levels of ATX in glioblastoma and endothelial cells at both mRNA and protein levels. Analysis of mRNA (Figures 1A,B) showed that glioma cell cells had higher expression ATX when compared to endothelial cells. We found that inhibition of ATX using PF-8380 in serum free media (Figure 2) reduced the clonogenicity of both the gliomas and the endothelial cells. These results indicate that inhibition of ATX is sufficient to prevent the formation of LPA and its downstream interactions with the LPA receptors as we had shown earlier (28).

We studied how ATX inhibition might influence cancer cell behavior *in vitro*. Noting previous work identifying ATX derived LPA as an essential stimulator of cancer cell invasion and migration (29), we hypothesized that the overexpression of ATX in glioblastoma is pivotal to invasiveness and motility observed at the cellular level. We performed migration and invasion assays in mouse GL261 and human U87-MG glioblastoma with combinations of PF-8380 and radiation. In wound healing assays, GL261 and U87-MG cells pretreated with ATX inhibitor prior to irradiation resulted in 17.9 and 33% decrease in cell migration in U87-MG and GL261 respectively (Figures 3A,B). In the tumor cell invasion assays, GL261 and U87-MG cells pretreated with PF-8380 prior to irradiation resulted in 50 and 35.6% decrease in cell invasion in U87-MG and GL261 respectively (Figures 3C,D). These results highlight a potential basis for the previous observations in the overall suppression of tumor development *in vivo* after ATX-inhibition (28). Furthermore, by illustrating significant reduction in chemotaxis following inhibition of ATX and irradiation, we have shown that ATX may play a major role in the development of radioresistance and recurrence of glioblastoma. The enhanced invasiveness and migration conferred by ATX activity may account for the extension beyond resection/irradiation margins and the subsequent evasion of therapy.

It has been shown that irradiating endothelial cells activates cPLA_2_ and leads to production of LPC, which is then converted to LPA by ATX (29–31). LPA in turn activates pro-survival pathways such as the PI3K-Akt pathway (21–24). Akt has been implicated with the radioresistance in glioblastoma (50) and is a downstream target of the commonly mutated RTK/RAS/PI3K pathway in glioblastoma (18). Apart from its effects on tumor growth, invasiveness, and angiogenesis, LPA is believed to play a major role in the mediation of pro-survival signaling in the tumor microenvironment (2). To determine the extent of ATX-derived LPA involvement in cancer cell resilience, we examined the effects of ATX inhibition on the activation of downstream pro-survival pathways. Inhibition of ATX resulted in reduced Akt activity in both U87-MG and GL261 after irradiation (Figure 4). This supports our observation of reduced colony formation, further linking ATX to cancer cell survival in the setting of radiotherapy. Our results also support the notion that LPA receptor activation by LPA represents a pathway for PI3K-Akt activation in glioblastoma.

While signaling at LPA receptors promotes the invasive and migratory characteristics of glioblastoma, LPA receptor activation in endothelial cells is known to promote neovascularization and tubule formation (28). However in the setting of radiation therapy, LPA receptor signaling is likely to result from activation of cPLA_2_. The important role of ATX in the formation and stabilization of blood vessels is highlighted in ATX^−/−^ knockout mice, which are embryonic lethal at ∼E9 and have severe vascular defects in the yolk sac (51). To study the effect of ATX inhibition on radiation-induced neovascularization, and to elucidate if LPA production by ATX is pivotal for the angiogenic response to radiation, we used a mouse tumor vascular window model to study the changes in tumor vascularity in response to combinations of radiation and PF-8380. We found that radiation triggered a slight increase in neovascularization, however pre-treatment with PF-8380 resulted in significant reduction in vascularity (Figure 5). The action of LPA generated by ATX on the tumor-endothelium appears to be critical for the normal response to radiation. This supports previous work where concurrent inhibition of ATX and LPA receptors was shown to reduce endothelial cell invasion, migration, and survival post-irradiation (28). However, this also indicates that inhibition of ATX may be sufficient to stifle angiogenesis in radioresistant gliomas. This observation, together with previous studies linking intracellular LPC to neovascularization in irradiated tumors, presents new relevance of LPA receptor mediated signaling downstream of cPLA_2_ and ATX activation in the vascular response.

In light of the anti-angiogenic effects of ATX inhibition observed in the vascular window model, and studies linking the overexpression of ATX in glioblastoma to its overall pathology (1, 30), we hypothesized that inhibition of ATX mediated LPA production in the tumor microenvironment would delay the tumor growth. We used a syngeneic mouse GL261 brain tumor model to evaluate if ATX inhibitor PF-8380 could serve as a therapeutic modality. Mice treated with PF-8380 prior to irradiation significantly delayed tumor growth when compared to mice treated with radiation alone (Figure 6). When compared to the untreated mice (DMSO), it took 12 additional days to attain a tumor volume of 7000 mm^3^ when the mice were treated with irradiation alone. When compared to irradiation alone it took eight additional days to attain a tumor volume of 7000 mm^3^ when the mice were treated with PF-8380 and irradiation. Mice when treated with PF-8380 and irradiation took 20.8 additional days for the tumor to attain a volume of 7000 mm^3^ when compared to untreated mice. Mice treated with ATX inhibitor alone did not show significant delay in tumor delay compared to untreated control. Mice treated with PF-8380 alone delayed tumor growth by 1.4 days to reach a tumor volume of 7000 mm^3^ compared to untreated control, suggesting that the role of ATX in tumor growth is linked to the radiation stress response (22, 46). These results indicate that the combination of ATX inhibition and irradiation can suppress tumor growth, thereby highlighting ATX as a molecular target in the radiosensitization of brain tumors.

In summary, the findings of this study suggest that ATX plays a pivotal role in translating the activation of cPLA_2_ by ionizing radiation into responses observed in the glioblastoma and endothelium. We found that inhibition of ATX with PF-8380 resulted in reduced production of LPA and disruption of downstream effects on glioblastoma cells *in vivo* and *in vitro*. In this study, inhibition of ATX prior to irradiation was sufficient to delay tumor growth, inhibit cell invasion, migration, and neovascularization, while also reducing survival of cancer cells. These findings, together with previous work highlighting the importance of LPA mediated signaling in tumor growth and cell survival, identify ATX as a viable molecular target for the radiosensitization of glioblastoma and destruction of the tumor vascular network.

## Authors Contribution

Designed and conceived experiments: Sandeep R. Bhave, Jerry J. Jaboin, Dinesh Thotala, and Dennis E. Hallahan. Performed the experiments: Sandeep R. Bhave, Amanda G. Linkous, Rowan M. Karvas, Rama P. Kotipatruni, Andrew N. Hallahan, and David Y. A. Dadey. Contributed reagents/materials/analysis tools: Dennis E. Hallahan. Analyzed the data: Sandeep R. Bhave, Todd A. DeWees, and Dinesh Thotala. Wrote the paper: Sandeep R. Bhave, Daniel J. Ferraro, Dennis E. Hallahan, Dinesh Thotala, and David Y. A. Dadey.

## Conflict of Interest Statement

The authors declare that the research was conducted in the absence of any commercial or financial relationships that could be construed as a potential conflict of interest.
